# Towards a model-based integration of co-registered electroencephalography/functional magnetic resonance imaging data with realistic neural population meshes

**DOI:** 10.1098/rsta.2011.0080

**Published:** 2011-10-13

**Authors:** I. Bojak, Thom F. Oostendorp, Andrew T. Reid, Rolf Kötter

**Affiliations:** 1 Centre for Computational Neuroscience and Cognitive Robotics, School of Psychology, University of Birmingham, Edgbaston, Birmingham B15 2TT, UK; 2 Centre for Neuroscience, Donders Institute for Brain, Cognition and Behaviour, Radboud University Nijmegen (Medical Centre), PO Box 9101/126, 6500 HB Nijmegen, The Netherlands; 3 McConnell Brain Imaging Centre, Montreal Neurological Institute and Hospital, McGill University, 3801 University Street, Montreal, Quebec, Canada H3A 2B4

**Keywords:** neural population model, multimodal integration, simultaneous EEG and fMRI BOLD, brain dynamics, cortical connectivity, mean-field model

## Abstract

Brain activity can be measured with several non-invasive neuroimaging modalities, but each modality has inherent limitations with respect to resolution, contrast and interpretability. It is hoped that multimodal integration will address these limitations by using the complementary features of already available data. However, purely statistical integration can prove problematic owing to the disparate signal sources. As an alternative, we propose here an advanced neural population model implemented on an anatomically sound cortical mesh with freely adjustable connectivity, which features proper signal expression through a realistic head model for the electroencephalogram (EEG), as well as a haemodynamic model for functional magnetic resonance imaging based on blood oxygen level dependent contrast (fMRI BOLD). It hence allows simultaneous and realistic predictions of EEG and fMRI BOLD from the same underlying model of neural activity. As proof of principle, we investigate here the influence on simulated brain activity of strengthening visual connectivity. In the future we plan to fit multimodal data with this neural population model. This promises novel, model-based insights into the brain's activity in sleep, rest and task conditions.

## 1. Introduction

Non-invasive recording of human brain activity has a long history, beginning with the electroencephalogram (EEG) [1]. The EEG remains prominent both in research and in clinical practice [2] owing to its excellent time resolution, which allows, for example, the tracking of evoked potentials. In the meantime, functional magnetic resonance imaging (fMRI) based on blood oxygen level dependent (BOLD) contrast has become a standard for researching cognition [3,4], largely because fMRI BOLD can locate brain activity with millimetre accuracy. However, progress in neuroimaging is slowing, owing to fundamental restrictions in acquisition methods. For example, volume conduction limits the spatial resolution of EEG to the centimetre range, whereas fMRI BOLD relies on vascular changes with latencies of half a second or more.

A promising way forward lies in combining already available neuroimaging modalities [5–9]. Each modality provides a particular and distinct representation of the brain's state via its specific signal sources. For example, EEG relies on electrical and fMRI BOLD on haemodynamic sources, which relate to different aspects of neural activity, as we shall see. Furthermore, data from different modalities often have complementary characteristics. This is the case for EEG and fMRI BOLD concerning spatiotemporal resolution, as discussed above. Finally, it is possible to record EEG and fMRI BOLD simultaneously [10]. This avoids the question whether data obtained in different sessions really refer to the same brain state. Hence EEG and fMRI BOLD present a convenient test case for developing multimodal approaches. An example for the ‘added value’ that simultaneous EEG/fMRI can provide is the connection of fMRI resting-state networks to EEG cortical microstates recently discovered by Britz *et al.* [11] and Musso *et al.* [12] (cf. the commentaries by Laufs [13] and Lehmann [14]).

There are three basic approaches to multimodal integration [15], which are all ‘model-based’ in some sense. When using *converging evidence*, a researcher combines data argumentatively against a backdrop of established expert opinion. Clearly, such an implicit ‘human mind’ model is powerful, but qualitative and idiosyncratic. *Data fusion* combines the various recorded data directly using statistical methods. This is quantitative and repeatable, but implies some model of relations between signal sources. Such relations may be less simple than commonly assumed (e.g. [16]). *Computational modelling* makes explicit prior knowledge and assumptions through the process of model specification. In principle, this allows the fully objective assessment of theory in terms of a model fit to data. In practice, realistic models are often too complex for a comprehensive validation or unequivocal falsification. Nevertheless, the explication of theory through a computational model generally allows more rigorous testing.

We present here a complete model chain from neural activity to detector signal, based on a neural population model (NPM). Pioneered by Wilson & Cowan [17] and others, such neural population modelling approaches have attracted much attention—see the recent reviews by Deco *et al.* [18] and Coombes [19]. They can successfully describe epileptic seizures [20–23], evoked potentials [24,25], cognitive activity [26,27], drug effects [28,29] and—of particular significance for this Theme Issue—sleep [30–33]. NPMs for the magnetoencephalogram [34,35] and fMRI BOLD [36] are popular, in particular, when considering network dynamics [37]. Here, we use a discretization of an anatomically folded cortex, with activity propagation instantiated not with approximate partial differential equations (PDEs) [38,39], but by explicitly keeping track of all signal delays. This allows the introduction of realistic cortical connectivity. Similar approaches have been developed by Sotero *et al.* [40] and Valdes-Sosa *et al.* [41].

Our NPM has been introduced previously in Bojak *et al.* [42]. Here, we add considerable technical detail necessary for implementing such a model. The paper is organized as follows. The following section explains how we extract the head model from structural MRI. Section 3 explains our NPM and signal expression. Section 4 presents new results for variations of specific connectivity strength. We then conclude with a discussion and outlook.

## 2. The head model

### (a) Surfaces extracted from structural MRI

**Figure 1.**
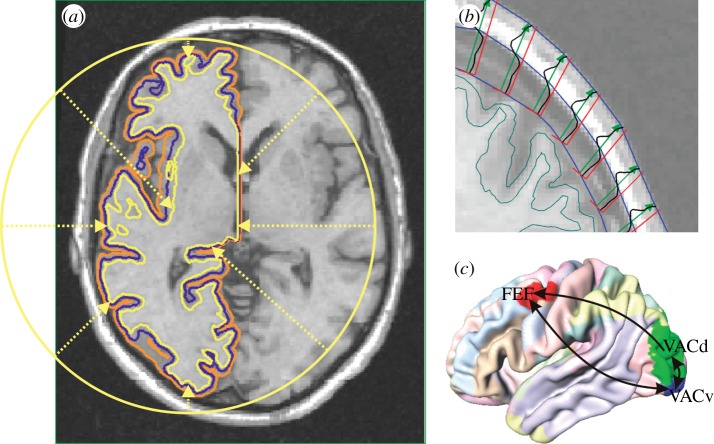
(*a*) Neural population model surface (blue) between the Civet interfaces of grey matter with white matter (yellow) and cerebrospinal fluid (orange). (*b*) Skull and scalp boundaries (blue) from intensity profiles (black) along outward vectors (green). (*c*) Visual connectivity used in this study. ‘Regional map’ areas are indicated by colours on an average cortical surface. FEF, frontal eye field; VACd and VACv, dorsal and ventral anterior visual cortex.

Surface approximations for the interfaces between grey matter (GM), cerebrospinal fluid (CSF) and white matter (WM) were obtained using the Civet software pipeline [43]. This involves a series of processing steps. Firstly, field non-uniformity artefacts are removed from *T*_1_-weighted structural images with the N3 algorithm [44]. *T*_1_-weighting is a standard MRI acquisition protocol that provides optimal intensity contrast between the tissue types of interest here. Secondly, the corrected images are normalized to stereotaxic space, and subsequently skull-stripped and classified into GM, WM and CSF. Thirdly, the GM/WM interface is constructed by deforming a spherical surface mesh subject to optimization constraints. Fourthly, Laplacian GM fields are computed, and CSF skeletons are constructed in deep sulci, as guides for an expansion of the GM/WM surface to the GM/CSF interface. The resulting meshes represent the borders of cortical GM, and we use an intermediate surface for modelling; see figure 1*a*.

To estimate volume conduction for EEG predictions, it is also necessary to obtain skull and scalp surfaces. Using the same images, and the obtained cortical surfaces as a starting point, *T*_1_ intensities can be sampled along rays extending outwards until a voxel containing ‘air’ is found, indicating the edge of the scalp. In such a *T*_1_-weighted image, skull tissue produces little signal, and thus its inner and outer boundaries can be determined as the edges of a ‘dip’ along the ray. Figure 1*b* illustrates this method. More details will be provided in a forthcoming publication.

### (b) Pruning the cortical mesh

The Civet mesh consists of 81 920 triangles with 40 962 vertices per hemisphere, but cortical folding can be represented faithfully with much fewer vertices. This is essential to reduce the NPM computation time, which scales linearly with the number of vertices. We will see below that ‘background connectivity’ scales roughly with the surface area, hence with the square of the number of vertices, and that longer edges between vertices reduce significantly the overall data transfer during the simulation. Thus, it is particularly advantageous to prune short edges. Furthermore, inspection of the Civet mesh shows that many small triangles are ‘wasted’ on relatively flat parts. In other places there appear unnatural ripples, typical evidence for ‘over-fitting’. These issues also are dealt with by removing short edges. Hence in each iteration of our algorithm, the shortest edge of the mesh is found. Subsequently, it is removed in a manner that we will detail next.

**Figure 2.**
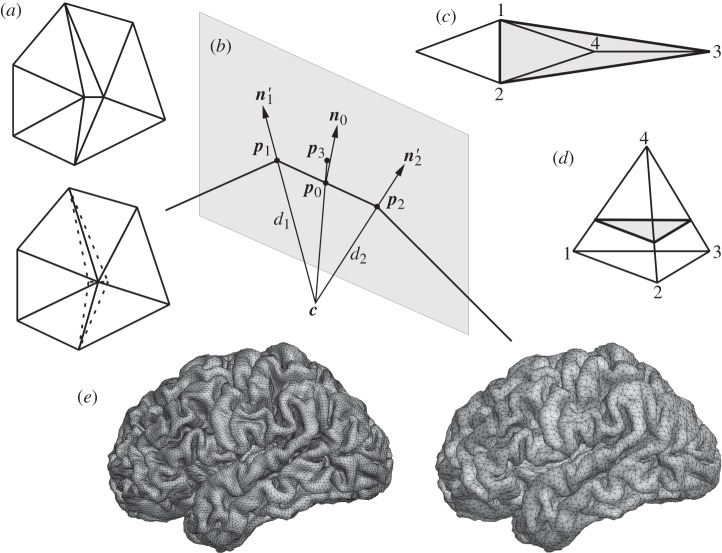
(*a*) Before (top) and after (bottom) pruning. (*b*) The bold edge between ***p***_1_ and ***p***_2_ is replaced by a vertex between ***p***_0_ and ***p***_3_. (*c*) If edge 1–2 is removed, triangles 2–3–4 and 1–4–3 collapse. The shaded region is replaced by the 1–2–3 triangle; or (*d*) a lifted one. (*e*) Cortical surface before (left) and after (right) pruning to a minimum edge length of 2.5 mm.

The two triangles that previously shared the shortest edge are removed, and their other two sides collapsed to a single edge terminating in a new vertex; see figure 2*a*. If the new vertex were to be positioned halfway on the former edge, the curvature of the cortex would be poorly preserved. Instead, the new vertex is positioned on a circle segment that approximates the surface (cf. figure 2*b*): ***p***_1_ and ***p***_2_ are the vertices to be removed, and ***n***_1_ and ***n***_2_ are the surface normals at these vertices, which are defined as the average over the area-weighted normals of the triangles they belong to. First 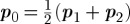 and 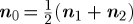 are computed. Next, ***n***_1_ and ***n***_2_ are projected onto the plane through ***p***_0_ that is spanned by ***n***_0_ and ***p***_1_−***p***_2_, resulting in ***n***_1_′ and ***n***_2_′, respectively. Point ***c*** is the intersection of the lines through ***p***_1_ with direction ***n***_1_′ and through ***p***_2_ with direction ***n***_2_′. Then *d*_*k*_ is defined as the distance between ***c*** and ***p***_*k*_ for *k*=1,2, and 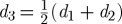. Finally, ***p***_3_ is defined on the line from ***c*** towards ***p***_0_ at a distance *d*_3_ from ***c***. The new vertex is placed at ***v***=***p***_0_+*b*(***p***_3_−***p***_0_) with a fixed parameter 0≤*b*≤1, where *b*=1 would be perfect for a sphere and *b*=0 for a plane.

The method fails for cases like edge 1–2 in figure 2*c*, since removing it would collapse two triangles onto each other. We refer to this as the ‘tetrahedron’, since the configuration resembles one if vertex 4 is not in the same plane, cf. figure 2*d*. Note that the base triangle 1–2–3 is *empty*, i.e. not itself part of the cortical surface. Tetrahedra are detected and removed by replacing them with single triangles; see figure 2*d*. We use a linear factor 0≤*l*<1, where *l*=0 indicates the edge base points 1, 2 or 3, and *l*=1 the peak 4. Thus, the tetrahedron is replaced by the base triangle for *l*=0 and with lifted ones for 0<*l*<1. While *l*>0 compensates better for loss of volume, it distorts the surrounding triangles. Figure 2*e* shows an original Civet mesh (left) pruned to a minimum edge length of 2.5 mm (right). We have used here *b*=1 and *l*=0. The pruned surface has 32 408 triangles with 17 208 vertices. The overall shape and surface area remain well preserved.

### (c) Parcellation and specific connectivity

Tractography based on diffusion MRI is popular for determining connectivity [45], but has significant drawbacks. Firstly, it is biased towards short-range connections and has problems where tracts are densely packed. Secondly, it does not determine the direction of activity propagation. Thirdly, it cannot find precise termination points in the GM. Data obtained through histological tract tracing methods are free of these problems, but only available from animal studies. We use here a connectivity matrix for macaque monkey (available on request), based on tracer data in the CoCoMac database [46], together with a ‘regional map’ (RM). This RM is a parcellation of cortex, which is sufficiently generalized to accommodate anatomical homology across primate species and uses area names that are widely recognized and convenient to use [47]. Since the Civet pipeline is designed to obtain optimal correspondence between individual vertices of meshes extracted from different human brains, it is sufficient to map macaque brain regions to a Civet mesh once. The assignment of vertex number to equivalent brain region stays the same for all Civet meshes. The RM was manually delineated onto a template macaque cortical surface (F99-UA1) and, by using two landmark-based deformations included in the Caret software [48], first mapped to the human PALS-B12 surface and then to our Civet template surface. The resulting parcellation is shown in figure 1*c*, and is also available on request.

## 3. Neural activity model and signal expression

### (a) The neural population model

**Figure 3.**
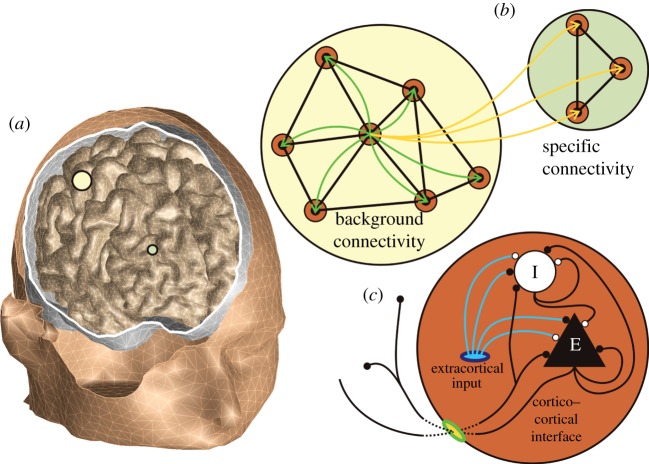
(*a*) Head model as extracted from structural magnetic resonance imaging. (*b*) For one vertex, the two kinds of long-range connectivity are illustrated. (*c*) The neural activity model with excitatory (black) and inhibitory (white) populations and connections.

Our software is flexible concerning the employed model of neural activity. We compute here at each vertex the NPM of Liley *et al.* [49], as extended by Bojak & Liley [28]. It contains one excitatory (e) and one inhibitory (i) NPM neuron, which represent those populations of real neurons whose coherent activity dominates the macroscopic signal of interest. For example, one can speculate that the EEG is largely due to pyramidal layer V ‘output neurons’, which form long dendritic bundles [50] that can act as dipolar current sources. The NPM neurons are locally connected to each other (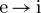, 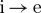) and to themselves (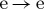, 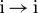); see figure 3*c*. NPM self-connections model real neurons of the same type connecting to each other.

The NPM consists of the following ordinary differential equations (ODEs):
3.1
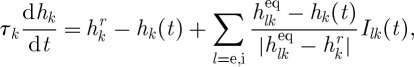

3.2
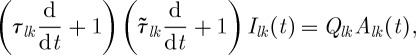

3.3
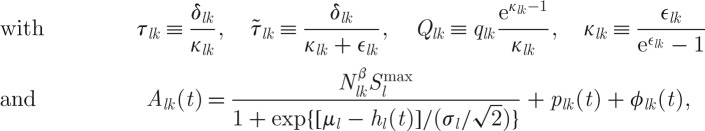

where *l*,*k*=e,i and *κ*_*lk*_|_*ϵ*_*lk*_=0_≡1 is continuous in *ϵ*_*lk*_. The model parameters could vary from vertex to vertex, but we choose here uniformly those of [51] and set *ϵ*_*lk*_=0 throughout.^1^ Equation (3.1) gives the response of the mean soma membrane potential *h*_*k*_ to a sum of post-synaptic potentials (PSPs) *I*_*lk*_. In the absence of input, *h*_*k*_ decays exponentially to 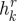 with characteristic time *τ*_*k*_. PSP impact is weighted, with a sign change at the Nernst potentials *h*^eq^_*lk*_. Equation (3.2) responds to a pre-synaptic Dirac pulse *A*_*lk*_(*t*)=*δ*(*t*) with an alpha PSP 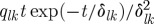 for *ϵ*_*lk*_=0, and a bi-exponential PSP 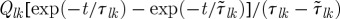 otherwise. *Q*_*lk*_ is proportional to the total charge transferred, *δ*_*lk*_ is the rise time to maximum PSP amplitude, and *ϵ*_*lk*_ prolongs the characteristic PSP decay time.

Equation (3.3) collects the sources for pre-synaptic spikes. The first term corresponds to the local firing rate, limited to 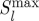, multiplied by the number of local synapses 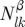. The second term, *p*_*lk*_, allows for extracortical input. Here, one could insert thalamocortical loops [52] or specific sensory inputs. We assume simply that extracortical input is noise-like in *p*_ee_ [28,49]. As a continuous average of neural signals, *p*_ee_ should be low-pass filtered. We use the spline variant of Catmull & Rom [53] with noise innovations as control points to construct such an input. This allows us to minimize the computational expense for random number generation and spline coefficient computation, which is important since the noise input is computed for every node independently. Catmull–Rom splines are unbiased, interpolating cubic splines with zero local tension and *C*^1^ (first derivative) continuity [54]. The following pseudo-code illustrates how one can interpolate *p*_ee_ at time steps *s*=0,1,2,…, where time *t*=*s* Δ*t*, from the Gaussian white noise rand_*n*_ sampled every *u*th time step.


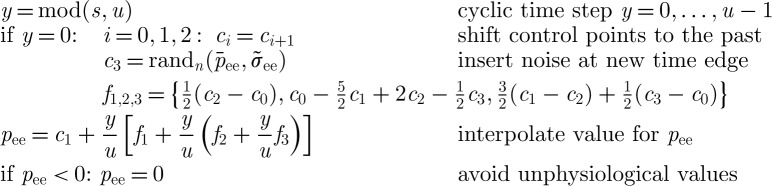


Setting up-sampling *u*=round[0.40449/(Δ*t* *f*_cut_)] achieves an intended −3 dB frequency, chosen here as *f*_cut_=75 Hz. The spline output has mean 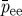 and standard deviation *σ*_ee_ if 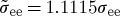.

### (b) Connectivity and propagation of activity

The third term of equation (3.3), *ϕ*_*lk*_, supports long-range connectivity. We distinguish two kinds; cf. figure 3*b*. Background connectivity is roughly isotropic, homogeneous and diminishes exponentially with distance [55–57], typically leading to waves of cortical activity [58]. This type of connectivity has been commonly used, since it can be approximated by PDEs [38,39]. Background connections from vertices *b* to a particular vertex *a* are modelled here by
3.4
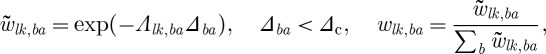

3.5
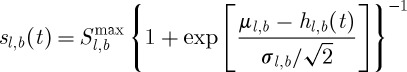

3.6
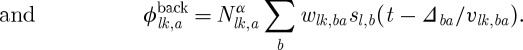

Background synaptic weights *w*_*lk*,*ba*_ sum to one and are multiplied by 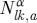, the number of synapses formed at vertex *a*. The firing rates *s*_*l*,*b*_ from vertices *b* arrive with delays *Δ*_*ba*_/*v*_*lk*,*ba*_, where *Δ*_*ba*_ indicates the distance along the cortical surface and *v*_*lk*,*ba*_ the conduction velocity; *Δ*_*ba*_ is estimated as the shortest path through the cortical mesh. We consider here only distances up to a cut-off *Δ*_*c*_, where 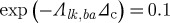, in order to limit the number of connections to those with significant impact. Long-range connectivity is here considered as exclusively excitatory (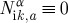), with characteristic decay (1/*Λ*_e*k*,*ba*_=2.5 cm) and cut-off (*Δ*_*c*_≃5.76 cm) distances in the right range for the loss of coherence measured with subdural electrodes [59]. Note that other synaptic footprints can be introduced simply by changing the functional form of 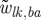.

Specific connectivity is implemented by adding further synaptic weights 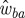:
3.7
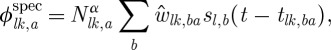

where the conduction delays *t*_*lk*,*ba*_ must be given. We will explain our estimation method below. For example, a specific synaptic weight 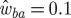 means that the added connectivity from vertex *b* to vertex *a* has 10% of the strength of the total background connectivity that *a* is receiving, since 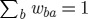. Specific connectivity can accommodate arbitrary connections between brain regions, and typically will be constructed according to some experimental connectivity matrix. Since the conduction delays *Δ*_*ba*_/*v*_*lk*,*ba*_ and *t*_*lk*,*ba*_ are not known with great accuracy, we discretize both internally as multiples of the time step Δ*t*=5×10^−5^ s. This makes delay bookkeeping much easier. The *ϕ*_*lk*,*a*_ represent averaged and hence continuous signals, for which we again use the spline approach detailed above. Let us consider *f*_cut_=1.6 kHz as sufficient, then up-sampling *u*=5 follows. Thus, we actually need to transmit *ϕ*_*lk*,*a*_ values only every *u*Δ*t*=0.25 ms. However, the spline needs four control points spaced by three time steps, hence we can only spline delays greater than 3*u*Δ*t*=0.75 ms. For *v*_ee,*ba*_=3 m s^−1^, the minimum allowed distance is *Δ*_*ba*_=2.25 mm. This demonstrates the connection between data transfer and shortest edge length: if we reduce the latter, then we must lower the up-sampling and hence transmit *ϕ*_*lk*_ more often.

For parallel computation, we divide up the cortical mesh between compute nodes. To minimize the communication overheads, we compile data into chunks for transmission between nodes. Assume a compute node 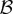 has been assigned vertices *v*_*b*_ with *b*∈*B*. A different node 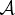 contains vertices *v*_*a*_ with *a*∈*A*. Call 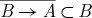 the list of vertices for which connection strengths *w*_*lk*,*ba*_ or 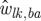, or both, are non-zero. The compilation of values {*s*_*l*,*b*_(*t*)} with 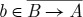 is what gets transmitted from 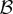 to 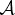. To simplify our explanation of how node 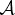 distributes the received data, we will assume that data are exchanged at every time step Δ*t*. In reality, only the spline control points are sent. This leads to a significant reduction of traffic, here by a factor *u*=5. Every vertex has its own ‘delay buffer’ for accumulating inputs, with a size set by the maximum (discretized) delay of incoming connections. A pointer indicating ‘current time’ advances in this buffer and at its end cycles back to the beginning. Distributing {*s*_*l*,*b*(*t*)_} now simply reduces to adding 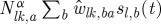 to the position *t*_*lk*,*ba*_/Δ*t* steps ahead of the ‘current time’ pointer (modulo the buffer length), and likewise for background connections. When the ‘current time’ pointer has advanced to a new position in the buffer, the sum of equations (3.6) and (3.7) will have been built up there. The buffer entry is hence added to the system as the current input *ϕ*_*lk*,*a*_, and then reset to zero, ready to accumulate input again.

**Figure 4.**
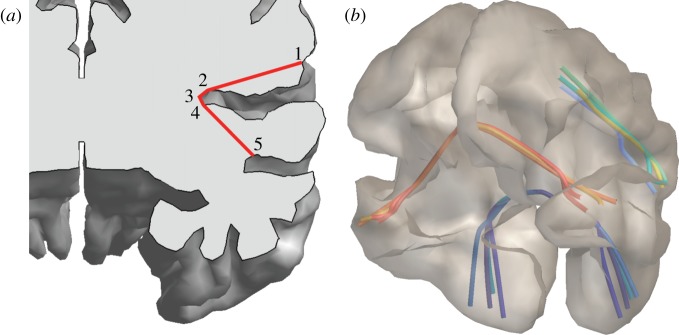
(*a*) Direct connections 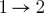, 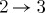, 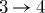 and 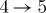 within the cortical volume are concatenated to the shortest route 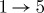. (*b*) Examples of shortest routes so determined.

The RM does not provide an estimate for fibre length and hence time delays *t*_*lk*,*ba*_. To calculate an estimate, we assume that vertices are connected by the shortest possible route, which minimizes conduction delays in the brain. Our algorithm first determines all those straight lines between pairs of vertices, which are completely contained within the cortical volume. This yields a network of allowed paths. At this stage any pair of vertices is connected at least via the surface edges, but ‘shortcuts’ through the volume generally exist. The optimal route is computed by means of a standard ‘all pairs shortest path’ algorithm like Floyd–Warshall [60]; cf. figure 4*a*. Examples of routes so determined are shown in figure 4*b*. For route finding, we prune the cortical surface further to only several thousand vertices. This limits the computation time for, and final size of, the distance matrix. Vertices of the NPM surface are then matched by Euclidean proximity.

### (c) EEG and fMRI BOLD signal sources

The details of extracting EEG and fMRI BOLD signals from the NPM simulation have been described in our previous publication; see Bojak *et al.* [42] for details. Electric dipole strength is here assumed to be roughly proportional to the average excitatory membrane potential *h*_e_ [61]. A volume conductor model with three compartments was constructed, representing the scalp (conductivity 0.2 S m^−1^), the skull (0.03 S m^−1^ [62]) and the inside of the skull (0.2 S m^−1^). For piecewise homogeneous volume conductors such as this, the boundary element method can be used to compute the EEG transfer matrix. We assume that fMRI BOLD is driven by glutamate release [63,64]. The neural drive *z* is hence proportional to the sum *f*_e_*A*_ee_+*f*_i_*A*_ei_ of excitatory inputs only, cf. equation (3.3). Values *f*_e_≃0.85 and *f*_i_=1−*f*_e_ represent the fraction of excitatory and inhibitory neurons, respectively. We implement a ‘balloon–windkessel’ model of haemodynamics at every vertex with four ODEs according to Friston *et al.* [65]. This predicts the local fMRI BOLD contrast *y*. For the results shown here, a baseline of resting activity is subtracted by hand.

### (d) Comparing voxel data with surface simulations

**Figure 5.**
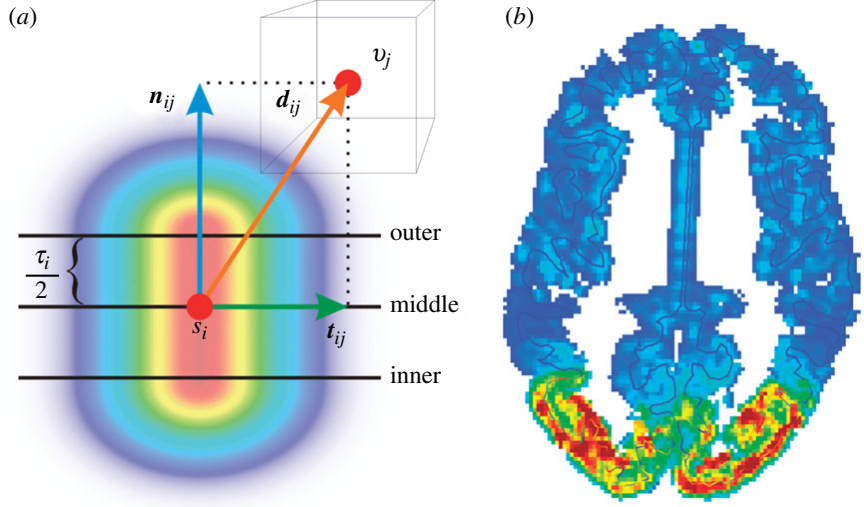
(*a*) Projection vectors for vertex *s*_*i*_ and voxel *v*_*j*_, and resulting weights from high (red) to low (blue). (*b*) Vertex to voxel projection of fMRI BOLD in horizontal section.

The prediction of voxel values from vertex ones must be informed by anatomy: in the normal direction of the surface we assume here that fMRI BOLD is roughly uniform across the cortical mantle. It is however straightforward to introduce a gradient, e.g. to weigh contributions from the pial surface more strongly. The weighting factors are then determined as follows (cf. figure 5*a*): if we designate ***d***_*ij*_ as the vector between a given vertex *s*_*i*_ and voxel centre point *v*_*j*_, then ***n***_*ij*_ is its projection onto the surface normal at *s*_*i*_; and ***t***_*ij*_ is a tangential vector uniquely defined by being orthogonal to ***n***_*ij*_ and coplanar with ***n***_*ij*_ and ***d***_*ij*_. Weights are computed by the Gaussian dispersion
3.8
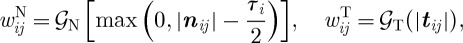

where 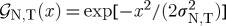, and we use *σ*_N_=2.0 mm and *σ*_T_=1.66 mm. The latter equates the Gaussian full width at half-maximum with the average edge length of our cortical surface (3.9 mm). Finally, the projected fMRI BOLD contrast *y*(*v*_*j*_) is
3.9
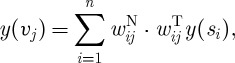

where *n* is the number of vertices in the surface mesh. A global normalization restores the original range of fMRI BOLD values. The same approach can be used as well to project fMRI BOLD voxel data onto surface vertices.

## 4. Results

The CoCoMac matrix provides basic information about connectivity strength by assigning values 0 (absent), 1 (weak), 2 (moderate) and 3 (strong) based on the reported histochemical staining. Here, we explore the relationship between CoCoMac's *anatomical* connectivity strength and the *effective* (causal) connectivity of our model, gauge the strength of specific versus background connectivity, and test the *functional* visibility of a cortical network consisting of the dorsal (VACd) and ventral (VACv) anterior visual cortex and the frontal eye fields (FEF). As can be seen in figure 1*c*, these areas form a triangle of connections, all with ‘moderate’ CoCoMac strength. We also include contra-lateral connectivity from every vertex to the vertex nearest to its mirror position in the other hemisphere. For the sake of simplicity, we employ only one universal 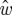 value for all specific connections. Varying this connection strength parameter will provide a basic test for how significant current experimental and theoretical uncertainties concerning (effective) connectivity are for model predictions of brain dynamics.

**Figure 6.**
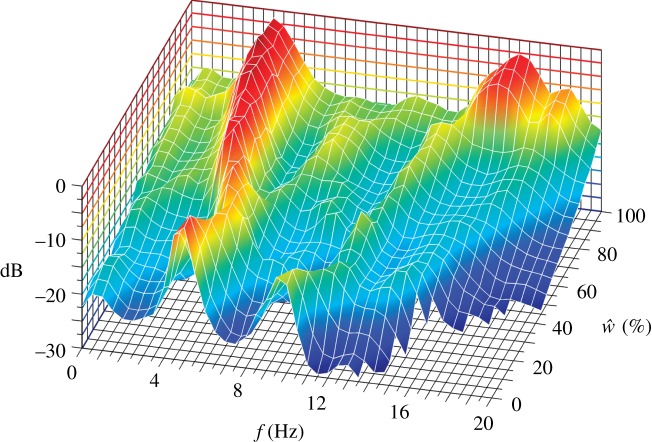
Dependence of the power spectral density of the ECoG on the strength of specific connectivity 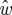. The power is shown in dB relative to the largest overall value.

We have simulated 10 s of brain time for 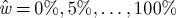, i.e. from no specific connectivity to one as strong as the background connectivity. The only input here is the noise shaped as described above, hence all dynamical features at frequencies well below *f*_cut_=75 Hz emerge from the system. The power spectral density of the excitatory mean soma potential *h*_e_ (simply called ‘ECoG’ henceforth) was computed for the last 4 s of brain time (2 s Hann window, 50% overlap), avoiding initial transients and averaged over all vertices. The result is shown in figure 6: a strong peak is evident in the delta/theta band, which drifts from 5 to 4 Hz for increasing 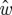. There is also a weak alpha peak at 9.5 Hz, which weakens further as it drifts to higher frequencies. Strikingly, upon moving from 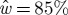 to 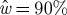, suddenly a strong beta peak appears at around 16 Hz, which for 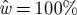 reaches three-quarters of the maximum power of the slow oscillation. The power of the slow oscillation itself by then has more than doubled. All the described features of the power spectra are due to self-sustaining oscillations, i.e. can be elicited also without the noise drive. We have tested that these results are stable for the simple (forward Euler) integration scheme used here by repeating the calculations with a five times smaller time step.

**Figure 7.**
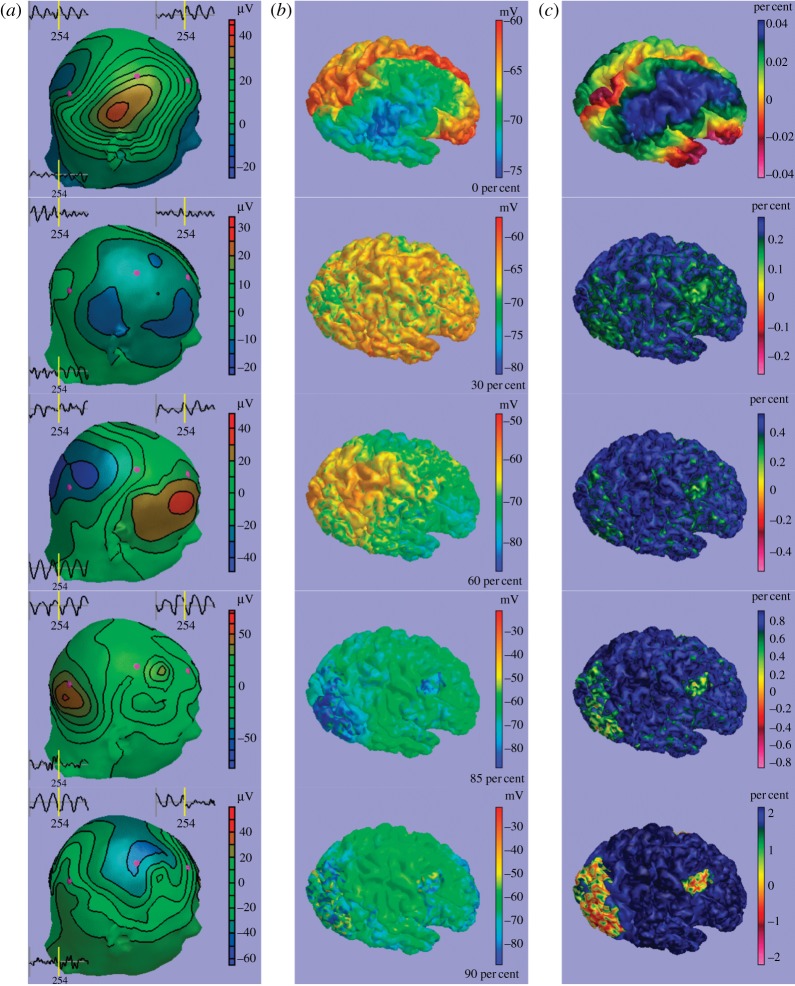
Predictions of (*a*) scalp EEG, (*b*) ECoG and (*c*) fMRI BOLD for different strengths of specific connectivity: 0% (top), 30%, 60%, 85% and 90% (bottom). See also the video in the electronic supplementary material.

In figure 7, we compare activity patterns for (*a*) scalp EEG, (*b*) ECoG and (*c*) fMRI BOLD for 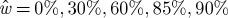, from top to bottom. In the EEG column, we also see recordings from three ‘electrodes’ indicated in purple, which show the last second of the simulation. Without specific connectivity, large waves of activity dominate. fMRI BOLD contrast gets sufficiently strong to be detected over experimental noise only between 30 and 60 per cent, and VACd, VACv and FEF also become somewhat visible there. The transition from 85 to 90 per cent appears less dramatic in the activity snapshots than in the power spectrum. However, for 90 per cent and higher we can easily identify the regions of our chosen cortical network, in particular with fMRI BOLD contrast. In summary, our variation of 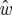 identified large parameter regions for which brain dynamics would appear qualitatively unchanged in simultaneous EEG/fMRI (30–85% and greater than 90%, respectively), but with the possibility for a transition between these regions induced by a small parameter change (from 85% to 90%).

## 5. Conclusions

In the present work and in Bojak *et al.* [42], we have outlined a complete simulation pipeline for forward predictions of simultaneous EEG and fMRI BOLD. It is based on a well-known NPM formalism [28,49], but uses a cortical tessellation with correct anatomical geometry in a realistic head model. Our software features a method for tracking conduction delays that allows the implementation of arbitrary corticocortical connectivity. We have explained here our pruning procedure for surface meshes, Catmull–Rom splining in noise generation and (multi-parallel) communication, the implementation of specific connectivity and finally the projection of surface fMRI BOLD predictions to voxel data. Furthermore, we have investigated the effects of strengthening specific connectivity. We found that a dynamical regime of dominant slow oscillations that allow the detection of functional networks with fMRI BOLD was qualitatively stable over a large parametric range (specific connectivity strength 30–85% relative to that of the homogeneous and isotropic ‘background connectivity’). However, a further small parameter increase (by 5%) would then result in the sudden appearance of additional strong beta oscillations. Consequently, for the time being it appears necessary to adjust connectivity strength carefully with respect to the resulting dynamics, since it is not guaranteed that predictions will be qualitatively similar for two different, but reasonable, parameter choices. We speculate that this difficulty generalizes beyond our current model, and suggest that any dynamical implementation of a connectivity matrix has to be accompanied by a careful exploration of the used connectivity strength.

With respect to frequency content, our simulations may already resemble stage II sleep. However, since our model oscillations are of purely cortical origin, lacking both realistic thalamic input and sleep cycle modulation, the current setup provides no direct insight into the mechanisms of sleep. Importantly, however, the NPM and connectivity can be easily modified or exchanged. Hence the sleep NPMs mentioned in §1 (cf. also Robinson *et al.* [66] in this Theme Issue) could be integrated with our software to allow realistic predictions of the activity of the sleeping brain as observed with (simultaneous) EEG and fMRI BOLD.

## Footnotes

1 The paper provides *γ*_*lk*_ and *Γ*_*lk*_ at *ϵ*_*lk*_=0, where *δ*_*lk*_=1/*γ*_*lk*_ and .
